# Replantation and Transplantation of Teeth

**Published:** 1884-02

**Authors:** 


### Replantation and Transplantation of Teeth.

Before the Academy of Medicine in Ireland (*Dublin Journal of Medical Science,* August, 1883), Dr. Theodore Stack read a paper on the replantation and transplantation of teeth. This subject, he stated, was first worthily introduced into surgical literature by John Hunter, in whose museum there is to be seen an immature cuspid transplanted into the comb of a cock with perfect success. Having fallen into disuse soon after Hunter's time, this method of treatment received a fresh stimulus from the practice at St. Bartholomew's of Mr. Coleman; and more recently Prof. Magitot, of Paris, has made a valuable communication on the subject to the International Medical Congress. Replantation may be found a useful therapeutic measure in—first, pulp exposed or nearly exposed with carious cavity extending under the gum; secondly, external violence knocking the teeth out; thirdly, accidental extraction; fourthly, obscure cases of neuralgia referred to sound teeth; fifthly, alveolar abscess, complicated or uncomplicated. It will be undertaken most frequently in cases of alveolar abscess. The primary cause of alveolar abscess is, in nearly every case, a putrefying pulp; a secondary cause may be a small portion of the tip of the root becoming necrosed by the abscess after it has lasted a little while, dissecting off from the part the periodontal membrane. Magitot proposed extraction of the tooth, resection of any necrosed part, and the replantation, and claimed a success of 92 per cent. Mr. Coleman proposed to fill the root antiseptically. This method appeared to fulfill the indications most fully, and some of Mr. Coleman's failures must be attributed to his dipping the tooth in too strong carbolic acid before replacement. Out of a table of some thirty cases made out by Mr. A. W. W. Baker and Dr. Stack from their private and hospital practice—all of which were successful—a large number had been treated by resection of the root, filling the root with creosote and iodoform, and free incision into alveolar abscess. Referring to the liability of these teeth to absorption—a danger mentioned by Tomes, Coleman, Nilhod, and others—Dr. Stack stated he believed this danger only applied to teeth which had been so treated when out of the mouth as to cause death of the periodontal membrane, either bv too long delay or by the use of some too strong chemical agent. It was not due to rending of the alveolar connections, for admittedly teeth violently knocked out and quickly replanted nearly always succeeded; nor was it due to placing foreign material in the pulp chambers and canals, for Dr. Stack was proud to say that in cases of teeth pivoted by two of their oldest dental surgeons—Mr. Robert Moore and Mr. Daniel Corbett—it was no uncommon occurrence for the roots to last twenty or thirty years. In the museum of the Dental Hospital of Ireland, there was a specimen of a pivot tooth presented by Mr. Corbett which had lasted thirty-seven years. In the allied operation of transplantation, when the scion tooth was always perfect, it is still undecided whether the pulp should be exterminated or not. Mr. A. W. W. Baker, Mr. Abraham, and Dr. Stack were, he believed, the first who had established by actual microscopical examination in the human subject that the pulp chamber in the scion tooth could after replantation again enclose living contents. This was a possible, perhaps a probable, result, but by no means a universal one. Dr. Stack believed that the operation of transplantation was likely to grow in favor, especially in hospital practice, where the patients were unable to pay for good artificial dentures.

